# *piggyBac* Transposon-Mediated Transgenesis in the Apicomplexan Parasite *Eimeria tenella*

**DOI:** 10.1371/journal.pone.0040075

**Published:** 2012-06-29

**Authors:** Huali Su, Xianyong Liu, Wenchao Yan, Tuanyuan Shi, Xinxin Zhao, Damer P. Blake, Fiona M. Tomley, Xun Suo

**Affiliations:** 1 National Animal Protozoa Laboratory and College of Veterinary Medicine, China Agricultural University, Beijing, China; 2 Institute for Animal Health, Compton, Berkshire, United Kingdom; 3 Royal Veterinary College, Pathology and Infectious Diseases, North Mymms, Hertfordshire, United Kingdom; Mayo Clinic, United States of America

## Abstract

*piggyBac*, a type II transposon that is useful for efficient transgenesis and insertional mutagenesis, has been used for effective and stable transfection in a wide variety of organisms. In this study we investigate the potential use of the *piggyBac* transposon system for forward genetics studies in the apicomplexan parasite *Eimeria tenella*. Using the restriction enzyme-mediated integration (REMI) method, *E. tenella* sporozoites were electroporated with a donor plasmid containing the enhanced yellow fluorescent protein (EYFP) gene flanked by *piggyBac* inverted terminal repeats (ITRs), an *Asc* I-linearized helper plasmid containing the transposase gene and the restriction enzyme *Asc* I. Subsequently, electroporated sporozoites were inoculated into chickens via the cloacal route and transfected progeny oocysts expressing EYFP were sorted by flow cytometry. A transgenic *E. tenella* population was selected by successive *in vivo* passage. Southern-blotting analysis showed that exogenous DNA containing the EYFP gene was integrated into the parasite genome at a limited number of integration sites and that the inserted part of the donor plasmid was the fragment located between the 5′ and 3′ ITRs as indicated by primer-specific PCR screening. Genome walking revealed that the insertion sites were TTAA-specific, which is consistent with the transposition characteristics of *piggyBac*.

## Introduction

Avian coccidiosis caused by infection with one or more *Eimeria* species parasite incurs global economic losses of ∼£1,500 million annually [1]. Traditional measures of control including prophylactic chemotherapy are becoming increasingly ineffective because of the emergence of resistance to anticoccidial drugs [2]. While several live attenuated *Eimeria* vaccines are commercially available [3], there is an urgent need for more cost-effective vaccines. A better understanding of the biology of *Eimeria* parasites is essential for the development of new strategies for effective control of avian coccidia.

Recent progress in genomic and proteomic studies focused on *Eimeria* are now providing valuable insights into eimerian biology [4], [5]. Additionally, the development of protocols supporting genetic manipulation including transient and stable transfection of *Eimeria tenella* is beginning to provide complementary tools for functional genomic and transcriptomic analyses [6], [7], [8], [9], [10]. Nonetheless, despite these efforts functional genomic studies remain limited for *Eimeria* because of the lack of powerful and user-friendly molecular tools.

*piggyBac* transposable elements have been used for effective and stable transformation with a wide variety of organisms ranging from lower eukaryotes including *Plasmodium* [11], [12] to higher invertebrates like *Drosophila* [13] and mammalian vertebrates [14]. For random mutagenesis *piggyBac* has a better ability to non-preferentially integrate into the genome of *Drosophila melanogaster*, compared to the popular P-element [13]. However, *piggyBac* shows a high preference for integration into predicted transcriptional units in the apicomplexan parasite *Plasmodium berghei* [12], which suggests its potential for disrupting normal gene expression in the related apicomplexan parasite, *Eimeria tenella*. Importantly, high levels of transformation efficiency have been reported in mouse and human cells using *piggyBac* [14]. Combined, these characteristics suggest that *piggyBac* has the potential to allow functional genomic studies to be performed in *E. tenella* by generation of random gene mutations.

The *piggyBac* transposon was originally derived from the insect cell line TN-368 [15], [16]. Structurally, the 594 amino acid *piggyBac* transposase is flanked by 13 bp inverted terminal repeats (ITRs) [17], [18]. As a class II transposable element, *piggyBac* is found to cut and paste into TTAA target-site sequences [18]. Thus, the transposon was considered to be the typical member of the TTAA-specific *piggyBac* transposon family. The transposition function of *piggyBac* has been developed into a binary system which includes a donor plasmid that can carry a gene of interest or a selectable marker flanked by *piggyBac* 5′ and 3′ ITRs and a helper plasmid that encodes the *piggyBac* transposase [17]. Following consideration of the efficient and extensive use of the *piggyBac* binary system for transformation we decided to test its potential with the protozoan parasite *E. tenella* in this study.

## Results

### Microscopic Observation of EYFP-expressing Parasites

**Figure 1:**
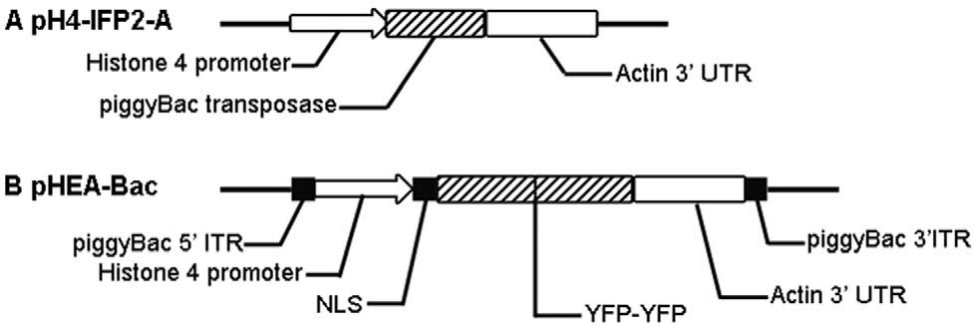
*piggyBac* transposon plasmids designed for transfection of *Eimeria tenella*. (A) The helper plasmid pH 4-IFP2-A. (B) The donor plasmid pHEA-Bac.

**Figure 2:**
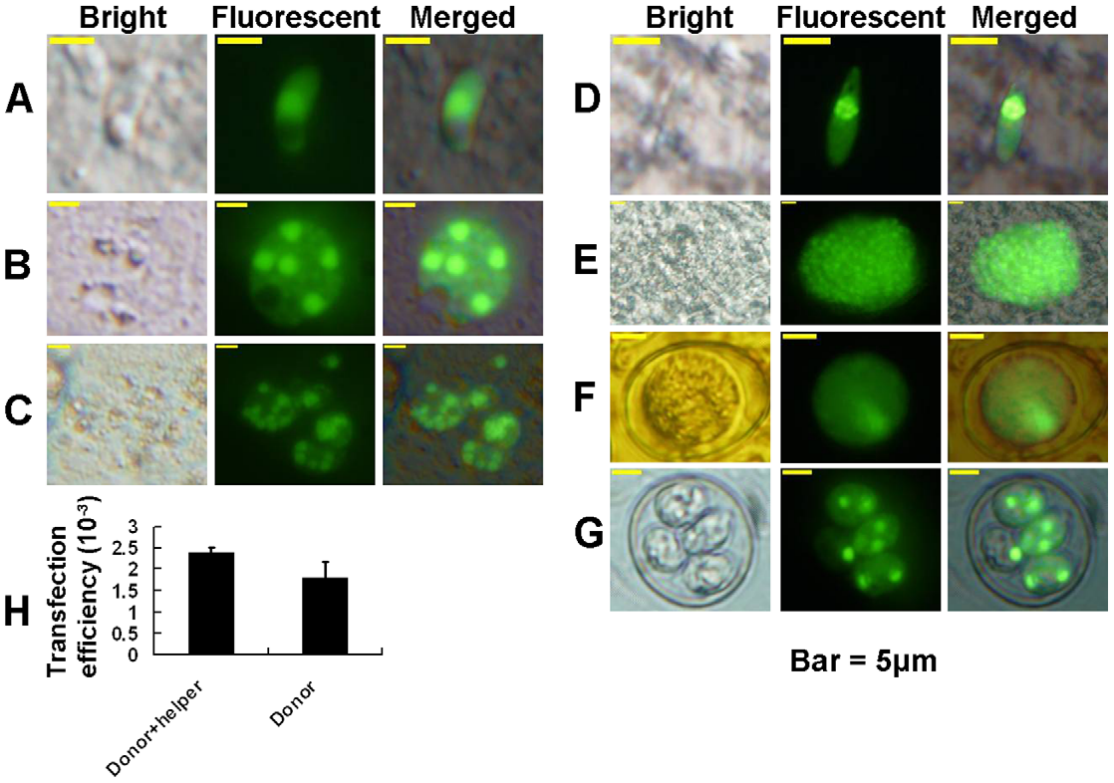
Transgenic fluorescent *Eimeria tenella* parasites. Sporozoite (A), early stage (B) and mature (C) first generation schizonts expressed EYFP predominantly in the parasite nuclei following transfection with the helper plasmid pH 4-IFP2-A and the donor plasmid pHEA-Bac. Late generation merozoite (D) and schizont (E), unsporulated (F) and sporulated (G) oocysts also presented fluorescent nuclei following transfection with pH 4-IFP2-A and pHEA-Bac. In the transfection efficient assays (H), fluorescent parasites in each well (3 wells per group) were counted at 36 h post transfection. The mean and standard deviations were analysed by the Student *t* test using the SPSS 13.0 software. The significance level was set at 0.05.

Parasites co-transfected with helper plasmid pH 4-IFP2-A (Fig. 1A, linearised, REMI) and donor plasmid pHEA-Bac (Fig. 1B, circular, non-REMI) were cultured on monolayers of primary chicken kidney cells (PCKCs). Fluorescent sporozoites (Fig. 2A) were detected 16 h post transfection. Further development of these transfectants including early stage **(** Fig. 2B **)** and mature first generation schizonts **(** Fig. 2C **)** was observed on the PCKC monolayer cultures. Microscopic examination of scraped chicken caecal mucosa sampled five days post-inoculation revealed fluorescent parasites undergoing later generations of schizogony **(** Fig. 2D and E **)**. Finally, fluorescent unsporulated oocysts **(** Fig. 2F **)** were purified from faeces excreted six to nine days post inoculation. Following sporulation, fluorescent sporulated oocysts were readily detectable **(** Fig. 2G **)**. As described previously [19], most of the fluorescent protein was targeted to the parasite nucleus under the influence of the 90-bp NLS. Transfection with circular donor plasmid pHEA-Bac and the restriction enzyme *Asc I* resulted in fluorescent parasites in the *in vitro* cultures at a similar level to the cultures infected with co-transfected (pHEA-Bac + pH 4-IFP2-A + *Asc I*) parasites (Fig. 2H) but did not result in any EYFP expression in parasites following *in vivo* infection of chickens. This is consistent with transient expression of EYFP from the donor plasmid, but with none, or negligible, non-homologous integration of the transgene in the absence of the helper plasmid.

### FACS Isolation of EYFP-expressing Oocysts from Successive Passages

**Figure 3:**
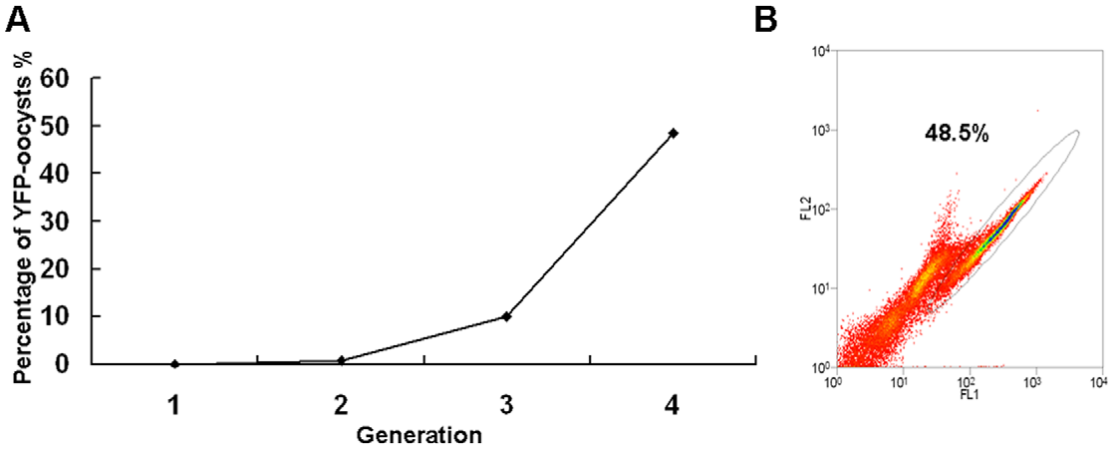
Isolation of fluorescent oocysts by FACS. (A) The percentage of fluorescent oocysts increased from 0.01% in the first generation to 48.5% in the fourth generation. (B) EYFP-expressing oocysts (48.5%) from the fourth generation.

Fluorescent oocysts produced following *in vivo* passage of sporozoites electroporated with the helper and donor plasmids were isolated by FACS and propagated by oral inoculation into four-day-old chickens. FACS analysis showed that the efficiency of transfection and transfected parasite survival was ∼0.01% after the first passage. The percentage of EYFP-expressing oocysts increased significantly during successive passage of FACS-sorted oocysts (Fig. 3A), rising to 0.8% and 10.2% of the second and third generations respectively. By the fourth generation 48.5% of the progeny oocysts were fluorescent (Fig. 3B).

### Characterisation of Exogenous Gene Insertion

**Figure 4:**
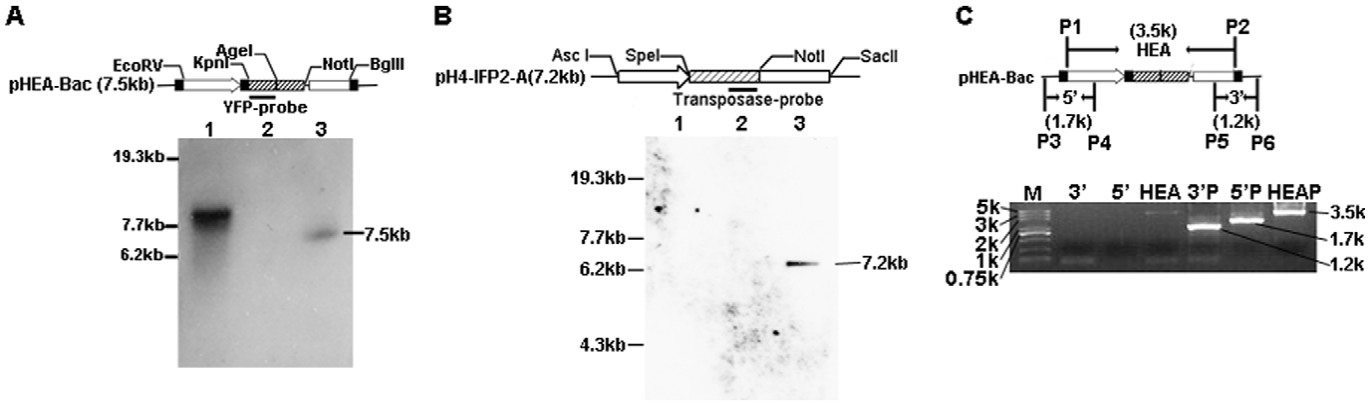
Confirmation of exogenous gene insertion. (A) Southern-blot hybridization using the EYFP gene as the probe. Lanes 1 and 2: transgenic and wild type *Eimeria tenella* genomic DNA, lane 3: donor plasmid pHEA-Bac (presenting EYFP as a positive control). (B) Southern-blot hybridization using the transposase gene as the probe. Lane 1: transgenic *E.tenella* genomic DNA, lane2: wild type *E.tenella* genomic DNA, lane 3: helper plasmid pH 4-IFP2-A (presenting transposase gene as a positive control). (C) PCR for confirmation of insertion of part of pHEA-Bac. The expression cassette “HEA” was only amplified from transgenic parasite genomic DNA and the 3′ and 5′ plasmid flanking sequences lying outside of the ITR sequences in pHEA-Bac were not detected. Lanes including “3′P”, “5′P” and “HEAP” represented positive controls (Amplicons from the plasmid pHEA-Bac).

To test the *piggyBac* insertion into the *E. tenella* genome we carried out southern-blot hybridization using part of the exogenous EYFP sequence as a probe. Genomic DNA extracted from the fourth generation of FACS-sorted parasites (Fig. 3B) was digested, fractionated electrophoretically and transferred onto positively charged nylon membrane. Hybridization with the EYFP-probe suggested that the exogenous EYFP gene had successfully integrated into the parasite genome (Fig. 4A). The low-intensity and disperse hybridization signals beyond the main hybridization fragment revealed a limited spread of insertions over the genomes of the transformed population. To exclude the possibility of genome integration of the helper plasmid pH 4-IFP2-A, southern blotting probe on the transposase gene was used to hybridize with the parasite genome DNA. No specific hybridization signal was detected in the transfected *E.tenella* genome, which suggested that there was no transposase gene inserted into the parasite genome (Fig. 4B).

We confirmed insertion of the expression cassette “HEA” from the donor plasmid pHEA-Bac into the *E. tenella* genome by locus-specific PCR. Using transfected *E. tenella* genomic DNA as template, amplification of the 5′ and 3′ expression cassette plasmid flanking sequences produced no bands, indicating the absence of the episomal form of pHEA-Bac. As expected, a specific band with size equal to the positive control was detected when we amplified the expression cassette “HEA” from transgenic *E. tenella* genomic DNA (Fig. 4C). Combined, these results indicated that only the part of the donor plasmid located between the 5′ and 3′ ITRs had been inserted, which is consistent with the integration characteristic of *piggyBac*.

### Identification of PiggyBac Integration Sites within the *E. tenella* Genome

Genome walking was used to identify the *piggyBac* insertion sites. Genomic DNA were extracted from the fourth generation of FACS-sorted parasites and the electroporated parasites (sporozoites and schizozoites) which had been cultured on the monolayers of PCKCs for 48 h post transfection. The *piggyBac* cassette left and right genomic flanking sequences were separately amplified and sequenced. Sequence analysis detected three different sites of integration at the left terminal region and seven different sites at the right terminal (Table 1). As expected, the *piggyBac* element had inserted in a TTAA target in all of the analyzed sequences. Moreover, sequence analysis performed using alignment with DNAMAN software revealed significant conservation among six insertion site sequences at the right end. For integration sites R1, R2 and R3 there was a 61-bp conserved sequence (CS1) adjacent to “TTAA”. For R4, R5 and R6 there was another 61-bp conserved sequence (CS2) at the integration site. We identified a single locus (Eimer-4441c03.p1k) following blast search of the L1 sequence. For L2 and L3, we could not find a highly similar sequence through blast search using the *E. tenella* omniblast server http://www.sanger.ac.uk/cgi-bin/blast/submitblast/e_tenella/omni, possibly due to the incomplete nature of the *E. tenella* genome sequence. At the right terminal we identified two loci as integration sites for R1 and R4 respectively (Eimer-4441c03.p1k and Eimer-642h02.p1k). Homologous sequences were not found for sites of R2, R3, R5, R6 and R7 when we performed blast searches as described previously, although a large number of repetitive sequences were identified with a low level of significance. Using sequences associated with the two genomic insertion points suggested that each mapped to a predicted non-coding region within ∼400 bp of a putative coding sequence. The conserved CS1 and CS2 sequences were both found at several loci without annotation in *E. tenella* databases of the Wellcome Trust Sanger Institute.

**Table 1:** Identification of genomic sequences flanking the piggyBac insertion sites in *Eimeria tenella*.

Integration site	Left end	Integration locus
L1	Aggacacact-TTAA-*piggyBac*	Eimer-4441c03.p1k
L2	Aacgcgggac-TTAA-*piggyBac*	Unknown locus 1
L3	Gtcgtattac-TTAA-*piggyBac*	Unknown locus 2
	**Right end**	
R1	*piggyBac*-TTAA-CS1^a^	Eimer-4441c03.p1k
R2	*piggyBac*-TTAA-CS1^a^	Unknown locus 3^c^
R3	*piggyBac*-TTAA-CS1^a^	Unknown locus 4
R4	*piggyBac*-TTAA-CS2^b^	Eimer-642h02.p1k
R5	*piggyBac*-TTAA-CS2^b^	Unknown locus 5^d^
R6	*piggyBac*-TTAA-CS2^b^	Unknown locus 6
R7	*piggyBac*-TTAA-cagggcgcaa	Unknown locus 7

a Conservative sequence 1 (CS1):

GTAATACGACTCACTATAGGGCGAATTGAAGCTGCCCTTTGGTGCAGATGAACTTCAGGGT.

b Conservative sequence 2 (CS2):

GAGGCGGAGTGTCCGCTGTTGCTGTGGGCAGAAAGAGGGCGGCGTAGAGAGGCATTTAGTG.

c R2 flanking region was revealed as an AT-rich DNA sequence.

d R5 flanking region presented in the form of tandem repeat sequences.

### Calculation of Average EYFP Insertion Rate

We extracted genomic DNA from transgenic *E. tenella* harvested from the fourth round of propagation to calculate the average EYFP cassette insertion rate using Real-time PCR with the fluorescence dye SYBR Green I. By comparing the number of EYFP copies with the total number of transgenic parasite genomes (real-time PCR for total parasite genomes corrected for the percentage that were found to be fluorescent by microscopy) there were on average ∼14 EYFP copies per expressing genome, indicating ∼7 insertions since the EYFP was presented as a tandem copy reporter.

## Discussion

Genomic resources for *Eimeria* species parasites are rare. Although great progress has been achieved in a whole-genome sequencing project for *E. tenella* through a combination of Sanger and next-generation sequencing [20], the genome assembly remains fragmented and many genes are yet to be identified or characterized. Strategies supporting genetic manipulation of the *E. tenella* genome are developing rapidly but direct methods for verification of gene function by targeted gene knockout remain elusive, complicated by low insertion efficiency and a requirement for serial *in vivo* passage to select and stabilize transgenic parasite lines. Another way to determine gene function is to employ forward genetic approaches to generate random gene mutations and characterize the resulting phenotypes. Thus, the *piggyBac* transposon system, which has shown high levels of efficiency in other organisms, may provide a powerful approach to study gene function in *E. tenella*.

We report here that the piggyBac transposon system functions in E. tenella by transposing exogenous sequences into TTAA sites. Previous studies have reported that *piggyBac* insertions showed a high preference of predicted transcriptional units in mice [14] and 5′ UTRs of genes in *Plasmodium falciparum* [11]. In our study only three of the ten genomic insertion flanking sequences could be mapped to the genome assembly, identifying two as yet unannotated loci predicted to be located in non-coding regions proximal to putative coding sequences. Several genomic insertion flanking sequences were found to be repetitive, featuring tandemly repeated sequences or a high AT content which may be associated with transposon-like elements and telomere-like repeats. In this study, we got limited insertions sites in *E.tenella*. This may be due to the much higher overall GC content of the *Eimeria* genome (http://www.sanger.ac.uk/Projects/E_tenella/). Another reason for the small number of cloned insertion sites may be the amplification of parasite through successive passage, in which EYFP-transgenic parasites with some selective or growth advantage were selected for outgrowth. For this reason, we performed three independent transfections in which the electroporated sporozoites were cultured on the monolayer of PCKC cells other than passaged through the chicken. As the plasmid contamination was difficult to be avoid in the freshly electroporated samples, we got only one insertion site at each terminal region (L3 and R7). So there are still much more work to be done to obtain new integration sequences to understand integration preferences in E. tenella. Although it is far from making any assumption, we identified the conserved sequences CS1 and CS2 at most of the minimal insertion sites except for L1, L2, L3 and R7. As expected, all the identified insertion sites do support the existence of a short TTAA *piggyBac* target insertion sequence.

Black et al. reported that REMI could dramatically increase the transfection efficiency of *Toxoplasma gondii* and enhance stable transfection [21]. In our study, REMI was used to increase the transfection efficiency of *E.tenell*a. But in *piggyBac* system, transposase gene should be discharged from parasite or else the piggyBac transposon could keep on jumping around the genome. In order to reduce the possibility of insertion of the helper gene into the parasite genome, we didn’t do any selection for transposase gene through the successive passages. Fortunately, genome hybridization with the transposase-probe produced no specific signal, which successfully excluded the possibility of genome integration of the *piggyBac* transposase gene.

Plasmids with relatively large sizes (>7 kb) often produce low transfection efficiencies *in vitro* and therefore reduce the potential for obtaining fluorescent oocysts from the first *in vivo* passage of electroporated sporozoites [22]. The current study also failed to obtain transfected oocysts post in vivo passage of sporozoites electroporated with the circular donor plasmid pHEA-Bac and the restriction enzyme *Asc I* (non-REMI enzyme for pHEA-Bac), indicating a very low level of non-homologous integration in the absence of the helper plasmid. However, when the *piggyBac* transposase were applied together with the circular donor plasmid, fluorescent oocysts were successfully obtained, indicating that the efficient transfection of the transposase plasmid could help increase integration of exogenous genes. While, the action of the AscI may or may not have helped with genome access. Increased integration facilitates in vivo selection of transfected oocysts, suggesting that *piggyBac* may be a practical molecular genetics tool for use in *E. tenella* transgenesis. Experiments performed in this report provide a first step toward development of an effective mutagenesis platform in the apicomplexan parasite *E. tenella* using the *piggyBac* system.

## Materials and Methods

### Parasites and Animals

All experimental procedures were approved by the China Agricultural University Animal Ethics Committee. (The certificate of Beijing Laboratory Animal employee, ID: 18086). The E. tenella Beijing (BJ) strain [7] was maintained, purified, and sporulated as described elsewhere [23]. Before transfection, purified oocysts were ground with a 7-ml glass tissue grinder to release sporocysts which were then treated with trypsin-bile solution (0.75% [w/v] trypsin and 10% [v/v] bile in PBS) to release sporozoites. Purification of sporozoites was carried out by DE-52 anion-exchange chromatography [24]. Freshly purified sporozoites were washed once in incomplete cytomix (10 mM K2HPO4:KH2PO4, pH 7.6; 120 mM KCl; 0.15 mM CaCl2; 25 mM HEPES; 2 mM EGTA; and 5 mM MgCl2) [6] and then suspended in the same buffer, supplemented with 2 mM ATP and 5 mM glutathione. In vitro cell culture of transfectants was carried out using primary chicken kidney cells (PCKCs) prepared from a ten-day-old chicken as described previously [19]. Arbor Acres (AA) broiler chickens were used for preparation of PCKCs and parasite propagation in vivo.

### Plasmid Constructs

The helper plasmid pH 4-IFP2-A (Fig. 1A) was constructed by cloning the *piggyBac* transposase coding sequence from pBSII-IFP2orf [25] using primers 5′-ACTAGTATGGGTAGTTCTTTAG-3′ that created a *Spe* I site and 5′-GCGGCCGCTCAGAAACAACTTTGGCACA -3′ that added a *Not* I site, under the control of the *E. tenella* Histone 4 promoter and the *E. tenella* actin 3′ untranslated region (UTR). The *Spe* I and *Not* I digested PCR product was then inserted into the *Spe* I and *Not* I digested vector pEtHEA [8].

The donor plasmid pHEA-Bac (Fig. 1B) was constructed by cutting the fragment containing the EYFP sequence under the control of the *E. tenella* Histone 4 promoter and incorporating a 5′ 90-bp nuclear location signal sequence (NLS) and a 3′ *E. tenella* actin 3′ UTR from pH-2E-A3′ [26] using *Eco* RV and *Bgl* II. The digestion product was cloned between the ITRs of the *piggyBac* element in the vector pXL-BAC II [25].

### Transfection

*Eimeria tenella* sporozoites were electroporated with the donor and helper plasmids using a restriction enzyme-mediated integration (REMI) method [22] where only the helper plasmid was subject to REMI. Briefly, 1×10^7^ freshly excysted sporozoites suspended in complete Cytomix [6] were mixed with 60 µg circular donor plasmid pHEA-Bac and 30 µg *Asc* I linearized helper plasmid pH 4-IFP2-A. Next, 200 U restriction enzyme *Asc* I was added to the sporozoite/plasmid mixture. Prior to transfection the total volume of the mixture was made up to 800 µl in a 0.4 cm cuvette (BioRad). Transfection was conducted by electroporating at 2.0 kV, 25 µF, resulting in a pulse time of 0.3–0.4 ms, with a Gene Pulser Xcell*™* Electroporation System (BioRad). Transfection with the donor plasmid pHEA-Bac alone was set as control. The transfected sporozoites were allowed to rest for 20 min at room temperature. For *in vitro* cultivation, electroporated sporozoites at 1×10^5^ cells/ml were added to monolayers of PCKCs and incubated at 41°C in a 5% CO_2_ incubator. PCKCs were grown in a 12-well multiwell plate (BD Falcon™) in 2 ml/well of DMEM (Gibco) containing fetal bovine serum (10% v/v), penicillin (200 U ml^−1^) and streptomycin (20 mg ml^−1^). For *in vivo* parasite propagation transfected sporozoites electroporated with the helper and donor plasmids were inoculated into four-day-old chickens via the cloacal route (2×10^6^ parasites per bird).

**Table 2:** Primers used during the application and confirmation of *piggyBac* mediated transfection of *Eimeria tenella*.

Primer ID	Sequence (5′–3′)	Purpose
P1	GATATCTGGTTAGGGCCTCAAGGGAA	Confirm donor insertion
P2	AGATCTACACTCCGCCTCTTAATCTTT	Confirm donor insertion
P3	CGGGTTTCGCCACCTCTGACT	Absence of cassette flanking sequence insertion
P4	CGCCGTTTAGGCTGCTTCTGC	Absence of cassette flanking sequence insertion
P5	GTAGGTATGGGAGGAGTGTAGTTGG	Absence of cassette flanking sequence insertion
P6	TAAATCGGAACCCTAAAGGGAGCCC	Absence of cassette flanking sequence insertion
P7	CGCCGATACCATCACGAACAACAACA	Genome walking nested primer
P8	GCTCCTCGCCCTTGCTCACCAT	Genome walking nested primer
P9	TGGTGCAGATGAACTTCAGGGT	Genome walking nested primer
P10	CGACCACTACCAGCAGAACACC	Genome walking nested primer
P11	AGTCCGCCCTGAGCAAAGACCC	Genome walking nested primer
P12	CGTTTGCAGCAGAGTAGTTCAT	Genome walking nested primer
P13	GTTGCAGCTAGGTGCGAGACA	*Eimeria* 5 S rDNA qPCR
P14	CAGCGCGTCCTCTCTACCAA	*Eimeria* 5 S rDNA qPCR
P15	TCCAGGAGCGCACCATCTT	EYFP qPCR
P16	ATGCCCTTCAGCTCGATGC	EYFP qPCR

### Microscopic Observation of EYFP-expressing Parasites

*E. tenella* parasites electroporated with both donor and helper plasmids, or with donor plasmid alone, were cultured *in vitro* within PCKC and observed at 16 h and 36 h post-electroporation using an inverted fluorescent microscope (IX 71, Olympus, Japan) with 488-nm excitation and 508-nm emission filters, under which the expressed yellow fluorescence appeared green. At 36 h, the numbers of green intracellular sporozoites, or developing first-generation schizonts, were counted (Liu et al, 2008). Transfected *E. tenella* parasites within *in vivo* mucosal scrapings prepared from the caeca five days post-inoculation, and progeny oocysts purified from faeces excreted six to nine days post-inoculation, were also examined by fluorescence microscopy.

### Selection of Stable Transformed Parasites

Positive selections of transfectants were performed by Fluorescence Activated Cell Sorting (FACS) using a MoFlo Cell Sorter (Dako Cytomation, Fort Collins, CO, USA) as described previously [8]. The sorted oocysts were sporulated and inoculated into four-day-old chickens for another round of selection. After four successive passages with selection, the transgenic *E. tenella* population was collected for genomic DNA analysis.

### Southern-blot Hybridization

Wild type or transgenic *E. tenella* genomic DNA was prepared from freshly purified sporozoites according to the Instruction Manual of TIANamp Genomic DNA Kit (TIANGEN, Beijing, China). *Bam* HI linearized donor plasmid pHEA-Bac was used as the positive control. For Southern blotting, 3 µg of genomic DNA was digested with *Nde* I and electrophoresed on a 0.8% (w/v) agarose gel (in 1× TAE, 60 V, 4 h). Size-fractionated DNA was blotted onto positively charged nylon membrane (Amersham Biosciences, NJ, USA) by capillary transfer and fixed to the membranes by u.v.-crosslinking for 10 min. The hybridization probe corresponding to the EYFP gene sequence was amplified with the primers 5′-ATGGTGAGCAAGGGCGAGGAGC-3′ and 5′-CTAAAGCTTCTTGTACAGCTC-3′ from the plasmid pHEA-Bac. The transposase gene probe was cloned from the plasmid pH 4-IFP2-A with the primers 5′-AATGGAATGCCTTATTTGGG-3′ and 5′-GTGTCCACTCCGCCTTTAGT-3′. Probe labeling, hybridization, washing and detection were carried out according to the manufacturer’s guide using a DIG High Prime DNA Labeling and Detection Starter Kit II as described previously [8].

### PCR Confirmation of Genomic Fragment Insertion

Primers specific for the donor plasmid expression cassette (containing the *E. tenella* Histone 4 promoter, EYFP gene and *E. tenella* actin 3′ UTR; P1 and P2, Table 2) located between the *piggyBac* 5′ and 3′ ITRs were used to confirm insertion of the donor plasmid pHEA-Bac vectored marker cassette into the *E. tenella* genome. Two further primer pairs were used to confirm the absence of plasmid DNA flanking the ITR sequences from the genome (P3 and P4, P5 and P6, Table 2). Briefly, the PCR conditions were 35 cycles of 94°C for 30 s, 55°C for 30 s, and 72°C for 3 min 30 s. PCR products were electrophoresed using a 0.8% (w/v) agarose gel (in 1× TAE, 80 V, 20 min).

### Genome Walking Through 5′ and 3′ ITR Flanking Regions

In order to identify the genomic *piggyBac* insertion sites, *piggyBac* 5′ and 3′ ITR flanking sequences were amplified and sequenced using a genome walking method (Genome Walking Kit; TaKaRa). For the fourth generation of FACS-sorted parasites, genomic DNA was prepared from freshly purified sporozoites as described previously. For the electroporated parasites, sporozoites or schizozoites were harvested 48 h post transfection by digestion of the PCKCs with 0.25% trypsin-EDTA for 5 min at 37°C. The harvested parasites and PCKCs were treated with DNase I (5 U/µg plasmid DNA) for 10 min at 37°C and washed five times with PBS (pH 7.2) [6] before genomic DNA was extracted with TIANamp Genomic DNA Kit (TIANGEN, Beijing, China). Two sets of three primers (P6, P7, P8 and P9, P10, P11) specific for the known pHEA-Bac vectored gene sequence were used successively in combination with the Arbitrary Primers (AP) provided by the kit. PCR products were gel purified, ligated into the cloning vector pEasy-T1 (TransGen Biotech, Beijing, China) and sequenced using the universal primers M13F & M13R. Analysis of the sequencing results was performed using DNAMAN software (Lynnon Biosoft, USA). Insertion sites were identified by similarity using BLAST searches with the *E. tenella* omniblast server http://www.sanger.ac.uk/cgi-bin/blast/submitblast/e_tenella/omni against the September 2010 genome assembly. Associated putative coding sequences were identified by BLASTX comparison of 5 Kb genomic sequence flanking the identified insertion site with the September 2010 *E. tenella* predicted proteins dataset.

### Genomic Insertion Quantification Using Real-time PCR

The average number of donor cassette insertions per parasite genome was determined using EYFP- and *Eimeria* genus-specific Real-Time PCR. Briefly, the total number of *E. tenella* genomes was determined for each test sample in triplicate using a genus specific Real-Time PCR assay targeting the 5 S rRNA coding sequence with primers P13 and P14 (Table 2) [27]. EYFP coding sequence copy number was evaluated in triplicate using primers of P15 and P16. Plasmids containing the EYFP or *E. tenella* 5 s rDNA sequence were constructed for use as DNA standards. Plasmid concentration was initially calculated using a spectrophotometer reading at 260 nm and 280 nm (NanoDrop 2000c, USA) prior to the preparation of ten-fold serial dilutions representing 10^6^–10^0^ copies of each plasmid. 2 µl of plasmid or transgenic *E. tenella* genomic DNA were used in each amplification reaction using the real-time PCR buffer Power SYBR® Green PCR Master Mix (Ambion, USA). Milli-Q water served as negative no template control. Amplifications were performed using Applied Biosystems 7500 Real-Time PCR System equipment and subjected to 50°C for 2 min, then 95°C for 10 min, followed by 40 cycles at 95°C for 15 sec and 60°C for 1 min. Figures produced by absolute quantification of target EYFP insert and 5 S rDNA host template copy number were used to calculate the average number of donor plasmid insertions per transgenic parasite genome based upon the total number of parasite genomes detected and the percentage found to be transgenic (i.e. fluorescent) by microscopy.
